# Supplementary material for: Exploring non-equilibrium processes and spatio-temporal scaling laws in heated egg yolk using coherent X-rays
Source: Nat Commun. 2023 Sep 11;14:5580. doi: 10.1038/s41467-023-41202-z (PMC10495384; doi:10.1038/s41467-023-41202-z)
Supplement: Supplementary file 1 — Supplementary Information [file 41467_2023_41202_MOESM1_ESM.pdf]

Supplementary Information: Exploring non-equilibrium processes and spatio-temporal scaling laws in heated egg yolk using coherent X-rays

Nimmi Das Anthuparambil[1,2*], Anita Girelli[3], Sonja Timmermann[2], Marvin Kowalski[2], Mohammad Sayed Akhundzadeh[2], Sebastian Retzbach[3], Maximilian D. Senft[3], Michelle Dargasz[2], Dennis Gutmüller[3], Anusha Hiremath[3], Marc Moron[4], Özgül Öztürk[2], Hanna-Friederike Poggemann[3], Anastasia Ragulskaya[3], Nafisa Begam[3], Amir Tosson[2], Michael Paulus[4], Fabian Westermeier[1], Fajun Zhang[3], Michael Sprung[1], Frank Schreiber[3] and Christian Gutt[2*]

[1]Deutsches Elektronen-Synchrotron DESY, Notkestr. 85, 22607 Hamburg, Germany.
[2]Department Physik, Universität Siegen, 57072 Siegen, Germany.
[3]Institut für Angewandte Physik, Universität Tübingen, 72076 Tübingen, Germany.
[4]Fakultät Physik/DELTA, Technische Universität Dortmund, 44221 Dortmund, Germany.

*Corresponding author(s). E-mail(s): nimmi.das.anthuparambil@desy.de; christian.gutt@uni-siegen.de;

This document complements the main text by discussing the details of the data analysis. Additional plots demonstrating the temporal evolution of the structure and dynamics of egg yolk gel during thermally induced non-equilibrium processes are also presented here.

1 Supplementary Note 1: Sample details

Egg yolk is a very complex assembly of proteins, phospholipids, and cholesterol in water. The egg yolk constituents are described in Supplementary Fig. 1. Yolk consists of approximately 50% water, 33% lipid, and 17% protein[1]. Around 119 types of proteins are discovered in egg yolk[1]. This complex natural assembly is stabilised via interactions between its constituents. Egg yolk can be separated into two fractions (plasma and granules) by moderate centrifugation [2]. The egg yolk-plasma is a translucent liquid (Supplementary Fig. 2c) which represents $\approx$ 80% of yolk dry matter. The major constituent of egg yolk-plasma is low-density lipoproteins (LDLs) (85% of yolk-plasma) which is a spherical core-shell nanoparticle with an average diameter of $\approx$ 30 nm, their size is varied between 17 to 60 nm. The core is made of triglycerides and cholesterol esters in a liquid state, and the shell is made of a mono film of phospholipids onto which apolipoproteins are attached as shown in Supplementary Fig. 1. The LDLs are water-soluble entities and the density of LDL is 0.982 g/cm^3. The hydrophobic interactions between the phospholipid tail and core lipids and hydrophilic interactions between the phospholipid head and water molecules stabilise this complex nano-assembly. LDLs contain 11-17% proteins and 83-89% of lipids (neutral lipids and phospholipids) [2].

2 Supplementary Note 2: Sample preparation

The hen egg was purchased from a local supermarket. The egg yolk was separated from egg white using a strainer as shown in Supplementary Fig. 2a. It was washed slowly in deionised water and rolled on a filter paper to remove the excess albumin [4]. The vitelline membrane, that separates egg yolk from egg white was punctured using a pipette tip and the yolk contents were filled in a falcon tube as shown in Supplementary Fig. 2b. In order to separate plasma and granule fractions, the fresh egg yolk was centrifuged with a speed of 5270 g for $\approx$ 36 hours at temperature 20 °C. The floating fraction is plasma which has a translucent yellow colour (Supplementary Fig. 2c). The extracted egg yolk-plasma was directly used for X-ray photon correlation spectroscopy (XPCS) measurements. The settled fraction is egg yolk-granule which has a light yellow colour compared to plasma. The egg yolk-granules were diluted with deionised water to a concentration of 910 mg/ml for the XPCS measurements. All samples were filled in a quartz capillary (diameter $\approx$ 1.5 mm), sealed with parafilm, which was used for measurements as depicted in Supplementary Fig. 2d. All samples were stored at $T = 5\,^\circ$C during the course of experiments ($\approx$ 3 to 4 days).

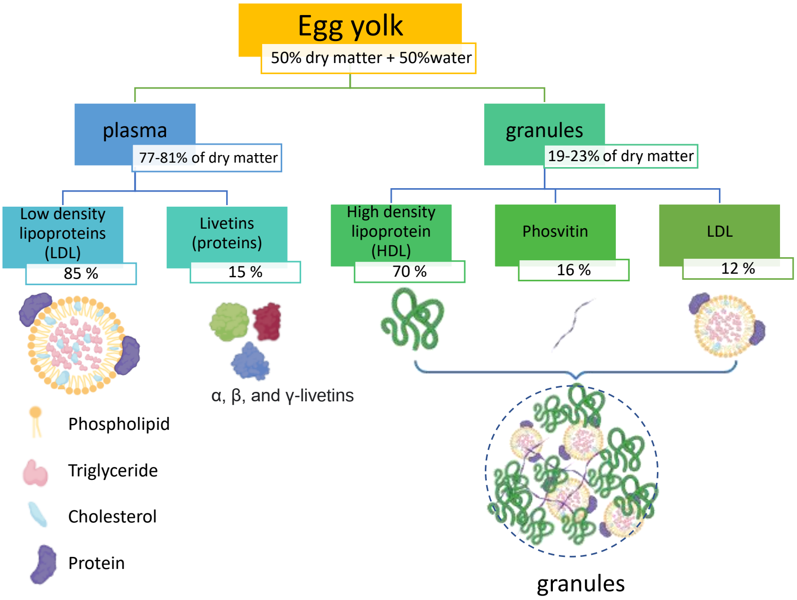

Supplementary Fig. 1 Constituents of hen egg yolk. The egg yolk can be separated into two major factions: egg yolk-plasma and egg yolk-granules via moderate centrifugation as described in the text. These factions are made of LDL, HDL, livetins, phosvitins, etc. (LDL: low-density lipoprotein, HDL: high-density lipoprotein). Parts of this schematic were created using Biorender [3].

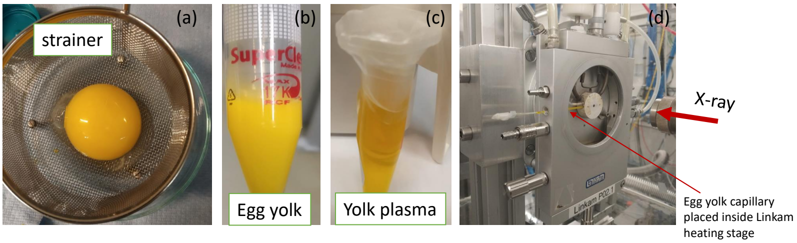

Supplementary Fig. 2 (a) The egg yolk is separated from the white using a strainer. (b) Egg yolk filled in a falcon tube. (c) Egg yolk-plasma separated from egg yolk. (d) The egg yolk sample was filled in a capillary, sealed with parafilm, and mounted in a Linkam heating stage for XPCS measurements at the P10 beamline.

3 Supplementary Note 3: Temperature calibration of Linkam heating stage

Since our measurements are very sensitive to absolute temperature values, we performed a temperature calibration of the Linkam heating stage. For this, a thermocouple was immersed inside a quartz capillary filled with egg yolk,

and placed inside the Linkam heating stage. The representative heating profile for a set temperature of 60 °C is shown in Supplementary Fig. 3a. The time needed to attain the final temperature from 22 °C, t_{heating}, is estimated from the heating profiles obtained with the thermocouple (Supplementary Fig. 3b) and these values are used in the paper.

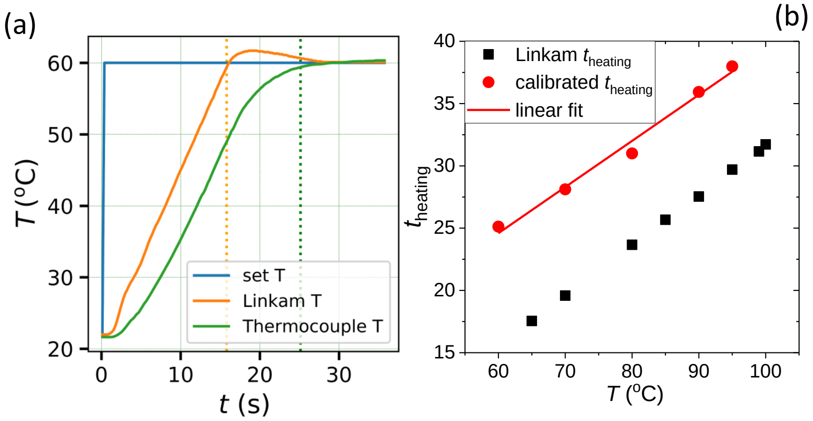

Supplementary Fig. 3 (a) The representative heating profile of Linkam heating stage for a set temperature of 60 °C. The orange and green vertical lines represent the t_{heating} measured by the temperature sensor inside the Linkam heating stage and the thermocouple inside the quartz capillary, respectively. (b) The calibrated t_{heating} compared to t_{heating} measured by the Linkam heating stage.

4 Supplementary Note 4: Details of XPCS and SAXS measurements

The XPCS measurements were performed in ultra-small angle scattering (USAXS) geometry at the P10 beamline of PETRA III, Hamburg. The results presented in this study were collected from different beamtimes, therefore the X-ray energy employed was 8.54 keV (for egg yolk and egg yolk-plasma) or 8.75 keV (for yolk-granules). The sample-to-detector distance was ≈ 21.2 m and an EIGER X4M detector with an active area of 162.5 mm x 155.2 mm (WxH) was used for recording XPCS data series. The available scattering wave vectors ($q = \frac{4\pi}{\lambda}\sin(\theta)$, λ: wavelength of X-ray, 2θ: scattering angle) range for this set-up was $\approx 0.005\,\mathrm{nm}^{-1}$ to $\approx 0.2\,\mathrm{nm}^{-1}$. The data were collected in 5-9 scans in which the total absorbed dose per scan is limited to 1 kGy. Each scan was performed on a fresh spot (separated by a distance of 200 µm). A typical scattering pattern collected using EIGER X4M is shown in Supplementary Fig. 4. The non-pixel areas (the red stripes in Supplementary Fig. 4) and the beam stop areas are masked in the data analysis. The data extraction and analysis were performed using a Python-based analysis package - Xana [5], and a MATLAB-based XPCSGUI provided by the P10 beamline. The structural information is obtained by extracting $I(q)$ profiles as a function of waiting time. Further, the dynamical information was accessed by calculating two-time correlation functions (TTCs).

Apart from XPCS measurements in USAXS geometry, we also performed small-angle X-ray scattering measurements (SAXS) measurements at the P10 beamline, PETRA III, Hamburg and beamline BL2 of the DELTA synchrotron radiation source. The q-range of SAXS experiments at P10 beamline (PETRA III) is $0.016\text{-}1.18\,\mathrm{nm}^{-1}$, and that for BL2 beamline (DELTA synchrotron radiation source) experiments is 0.26- $3.3\,\mathrm{nm}^{-1}$. The sample to detector distance of 5 m, incident energy of 8.54 keV, beam size of $\approx 7\mathrm{x}16\,\mu\mathrm{m}^2$ and an EIGER X4M detector with an active area of 162.5 mm x 155.2 mm (WxH) was used for recording SAXS data series at the P10 beamline.

For SAXS measurements at the BL2 beamline of the DELTA synchrotron radiation source, we used incident energy of 12 keV and beam size of $0.6\mathrm{x}0.6\,\mathrm{mm}^2$. The scattering data were collected by a MAR345 image plate detector with a constant exposure of 60 s. For temperature measurements, the samples were filled into borosilicate capillaries. In-situ heating measurements were performed by exploiting a dedicated temperature cell.

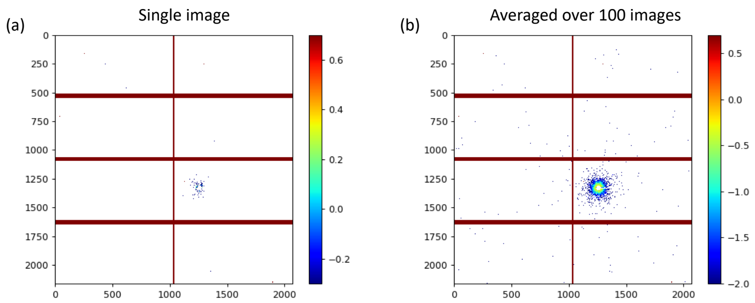

Supplementary Fig. 4 A representative scattering pattern (log $(I(q))$) from egg yolk samples collected using EIGER X4M detector. (a) A single image with an exposure time of 0.01 s, (b) averaged over 100 images. The x and y-axes represent the pixel number of the detector and the scale bar shows the logarithm of the scattering intensity of the pixels. The red stripes are non-pixel areas.

4.1 Background subtraction of scattering profiles

We utilised scattering data from deionized water (DI water) for background subtraction. As depicted in Supplementary Fig. 5, the buffer background is approximately two orders of magnitude less than the yolk data.

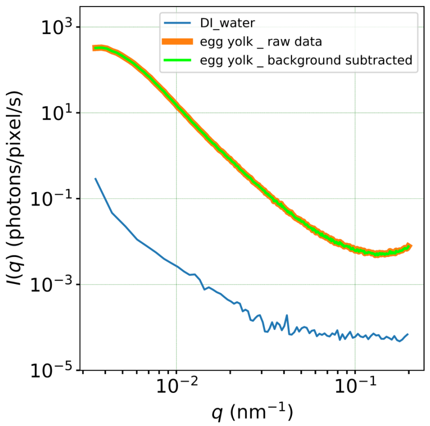

Supplementary Fig. 5 Comparison of raw and DI water subtracted $I(q)$ of the egg yolk. The background is approximately two orders of magnitude less than the yolk data.

4.2 Extraction of dynamical parameters

To follow a non-equilibrium process, it is necessary to get access to time-resolved dynamical parameters. The time-resolved sample dynamics is extracted by calculating the two-time correlation functions [6–17] using the relation,

$$C(q, t_1, t_2) = \frac{\langle I_p(q, t_1)\rangle \langle I_p(q, t_2)\rangle}{\langle I_p(q, t_1) I_p(q, t_2)\rangle}, \tag{1}$$

where I_p is the intensity at pixel p, $\langle ... \rangle$ denote the average over pixels in the q-range of $q \pm \delta q$ ($= 5.5 \pm 0.25\,\mu\text{m}^{-1}$ is used in this study), t_1 and t_2 are two time labels. The absolute waiting time is estimated as $t'_\text{w} = t_2$), and the relative time $t = |t_1 - t_2|$ increases away from the diagonal in the horizontal direction.

In Supplementary Fig. 6, a representative TTC is shown which contains information on the relaxation time, τ of the system under consideration. A bicubic interpolation is applied to all TTCs presented in the Supplementary Information to improve the image quality. The width of the bright patch along the diagonal is directly proportional to τ and its divergence indicates a fast-to-slow dynamical evolution (Supplementary Fig. 6). In order to extract the temporal evolution of relaxation time as a function of waiting time, the TTC is divided into small sections in the horizontal direction as depicted in Supplementary Fig. 6. A horizontal section of TTC, $C(q, t+t'_\text{w}, t'_\text{w})$, is nothing but the intensity autocorrelation which is modelled using the relation [11, 14, 18, 19],

$$g_2(q, t, t'_\text{w}) = 1 + \beta\ |f(q,t)|^2 = 1 + \beta\ |\ \exp[-(t/\tau)^\gamma]\ |^2, \tag{2}$$

where $f(q,t)$, τ, β and γ are the intermediate scattering function, relaxation time, speckle contrast, and Kohlrausch-Williams-Watts (KWW) exponent[20], respectively.

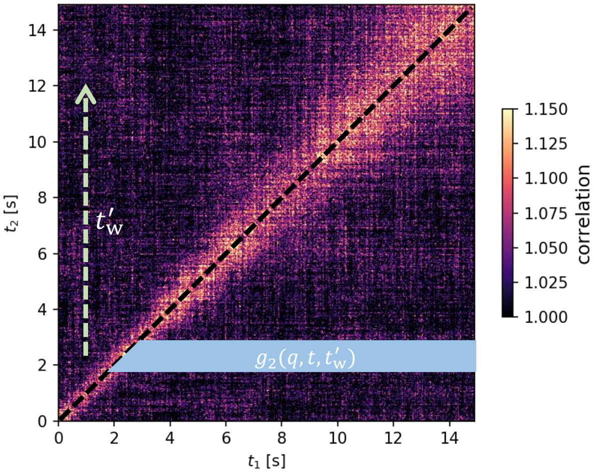

Supplementary Fig. 6 A representative TTC corresponding to a dynamically evolving system. The absolute waiting time is $t'_\mathrm{w} = t_2$. The light blue stripes represent sections of TTC extracted for estimating the $g_2(q, t, t'_\mathrm{w})$.

4.3 Beam effects on egg yolk samples

In X-ray scattering experiments, exposing the sample to X-rays over a critical dose can lead to radiation-induced damage. In XPCS measurements, X-ray beam-induced damage is characterised by following the changes in the structure and dynamics [19, 21–23]. Recent studies show that the beam-induced effects depend on both accumulated dose and dose rate [19, 23]. Hence, in order to avoid beam-induced artefacts in XPCS measurements, it is essential to find the critical dose and critical dose rate thresholds for a given sample system and perform later measurements below these limits. In order to estimate the critical dose and dose rate of egg yolk, we perform XPCS measurements at different incident X-ray intensities. The incident intensity of the X-ray beam is reduced to six different values by introducing silicon absorbers of different thicknesses in the X-ray path before the sample. The number of photons incident on the sample per second, Φ used here are listed in Supplementary Table 1. The dose, D absorbed by the sample is estimated using the relation [8],

$$D = \frac{\Phi E A_\mathrm{cof}}{d\, b^2\, \rho}, \tag{3}$$

where E, d, b, and ρ are the energy of the incident X-ray, the thickness of capillary (1.5 mm), beam size (100 µm) and density of egg yolk (1.028 g/ml [24]). The attenuation coefficient of egg yolk $A_\mathrm{cof} \approx 0.74$ is estimated as the fraction of transmitted intensity to incident intensity, which are measured using a silicon pin diode. The estimated values of the dose rate corresponding to six different values of Φ are depicted in Supplementary Table 1.

Incident photons, Φ (x 10^9 photons/s)	Dose rate (kGy/s)
0.06	0.004
0.37	0.025
0.69	0.046
2.34	0.155
27.16	1.8
92.48	6.129

Supplementary Table 1: Details of dose rate used for estimating beam damage.

First, we investigate the beam-induced effects on structural changes. In Supplementary Fig. 7 the temporal evolution of USAXS profiles collected at different incident intensities is shown. The scattering profiles are normalised with respect to the first profile, $I_0(q)$, and shown in Supplementary Fig. 7 middle row. Clearly, for a dose rate below 0.155 kGy/s, there is no change in scattering intensity for ca. 200 s. Beam-induced structural changes are observed when the dose rate increases to 1.8 kGy/s. The onset of beam-induced structural changes is indicated by the increase in the scattering intensity (5th column of 7). The changes are quantified by extracting the area (Area = $\int_{0.17}^{0.02} I(q)\,\mathrm{d}q$) under the $I(q)$ in the range 0.02 to 0.17 nm^{-1}. The 'Area' normalised with respect to the initial value is depicted in Supplementary Fig. 8a. Interestingly, the normalised area increases after a deposited dose of ≈ 40 kGy.

Next, we focus on the changes in the dynamical parameters of the sample. The representative TTCs extracted for XPCS scans with different incident intensities are shown in Supplementary Fig. 7 bottom row. Clearly, we observe equilibrium dynamics in TTCs until a dose rate of 0.155 kGy/s for ca. 200 s. To quantify the changes in dynamics, we extract autocorrelation functions and model them using Eq. 2. The extracted τ values are depicted in Supplementary Fig. 8b. For dose rates until ≈ 0.155 kGy/s, the dynamics measured in egg yolk samples are not hampered by any beam effects for a total dose of 8 kGy. Hence we perform all XPCS measurements within these limits. Specifically, all measurements presented in this study are performed using dose rates of 0.004, 0.025, and 0.046 kGy/s, and the absorbed dose per scan is limited to 1 kGy.

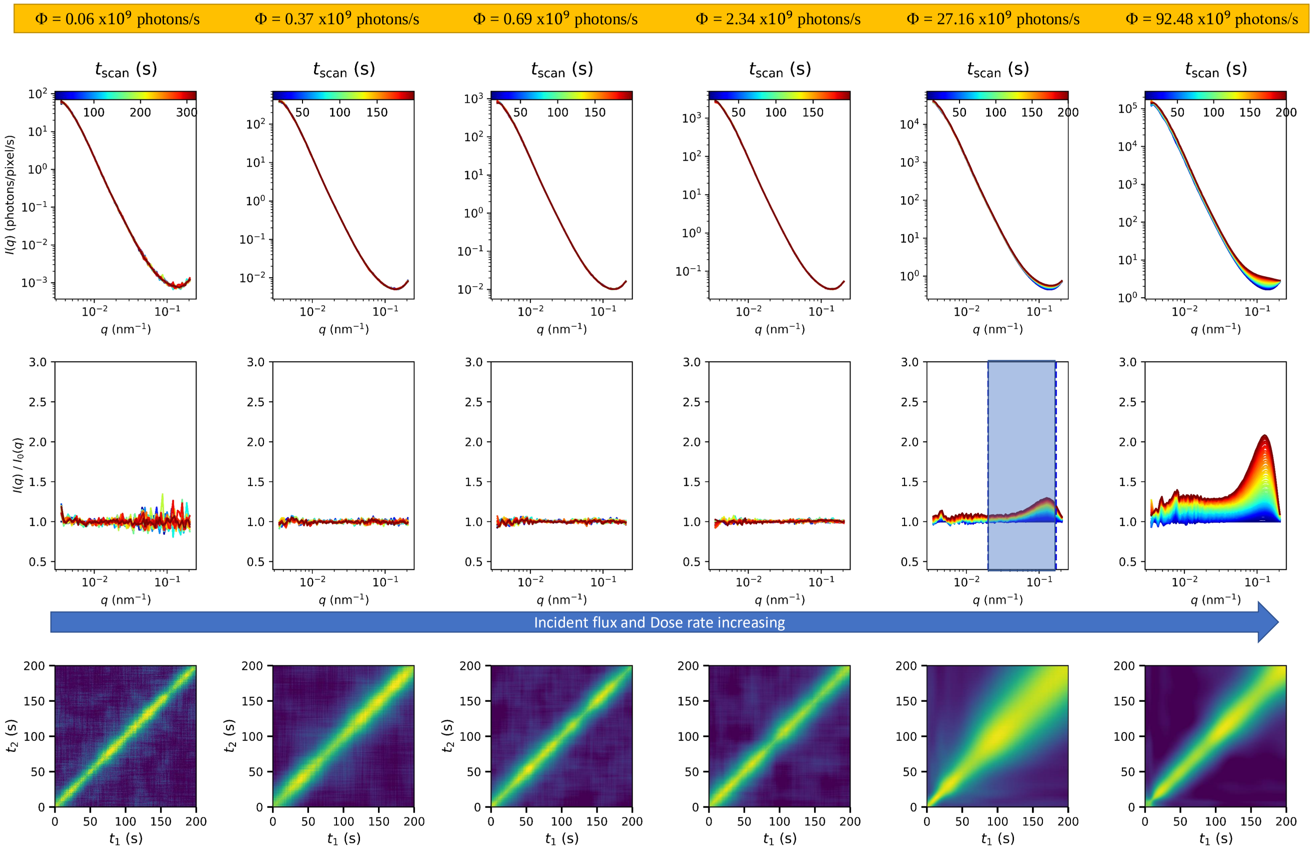

Supplementary Fig. 7 Top row: Temporal evolution of $I(q)$ as a function of q for different Φ as mentioned in the figure title. The scale bar represents the time elapsed in a scan (t_{scan}). Middle row: Normalised $I(q)$ profiles of the top row with respect to the first profile $I_0(q)$. The scale bar shown in the top row figures also applies to corresponding middle row figures. Here, any deviation (considering the noise in data) from $I(q)/I_0(q) = 1$ is considered as the change in the structure caused by the beam. The onset of structural changes is indicated by an increase in scattering intensity. With increasing total absorbed dose, the scattering intensity at high-q seems to change as depicted in the 5th plot of the middle row. The rectangle represent the regime $q \approx 0.02$ - $0.17\,\mathrm{nm}^{-1}$. Bottom row: TTCs extracted for various incident intensities as mentioned in the legend (at the top of the figure).

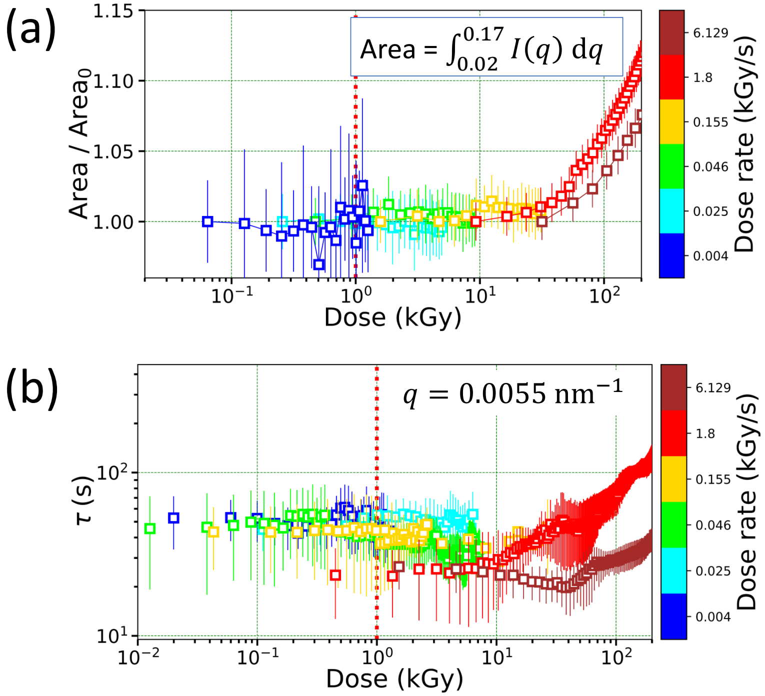

Supplementary Fig. 8 (a) The normalised 'Area' ($= \int_{0.17}^{0.02} I(q)\ \mathrm{d}q$) with respect to initial 'Area' (Area$_0$) as a function of accumulated dose. The error in 'Area' is estimated by considering the standard error in $I(q)$. For each dose rate, τ is estimated from 3-4 XPCS scans performed on fresh spots and their average is depicted in (b) and error bars indicate the standard deviation. The vertical dashed red line represents $D = 1$ kGy.

5 Supplementary Note 5: Structure of egg yolk, egg yolk-plasma, and egg yolk-granules at room temperature

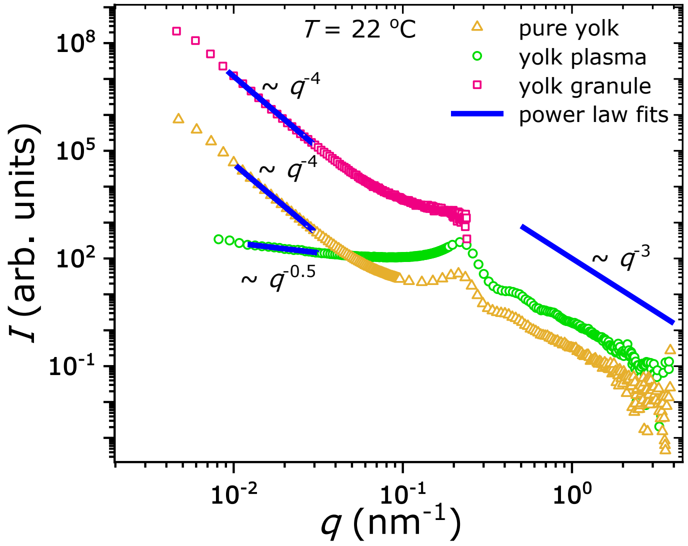

Supplementary Fig. 9 Comparison of scattering profiles of egg yolk, egg yolk-plasma, and egg yolk-granules. The USAXS profiles and SAXS profiles are stitched together for yolk and plasma samples. The curves are shifted along the y-axis for clarity.

The scattering intensity profiles contain information about the scatters in the given sample and are proportional to the electronic contrast of constituents and their volume. In order to decouple the contribution of individual components on the total scattering intensity from egg yolk, we perform scattering measurements on egg yolk factions: plasma and granules. In Supplementary Fig. 9, a comparison of scattering profiles of egg yolk, yolk-plasma, and yolk-granules is provided. The main features of SAXS profiles of the yolk are,

1. The Porod power law regime ($I(q) \propto q^{-4}$) below $q \approx 0.04\,\mathrm{nm}^{-1}$.
2. The structure factor peak at $q \approx 0.22\,\mathrm{nm}^{-1}$. From the structure factor peak position, we estimate an inter-particle distance of $\approx$ 30 nm, which is approximately equal to the reported size of yolk LDLs. Since $\approx$ 85% of yolk-plasma is constituted by yolk-LDLs, we assume that this value is approximately equal to the diameter of LDLs.
3. The higher order oscillations of structure factor in the high-q regime.

4. The asymptotic power law behaviour $I(q) \propto q^{-3}$ at high-q. This indicates the surface fractal nature of LDL particles with a fractal dimension of 3 [25].

On comparing the SAXS profiles of full yolk and yolk-plasma, it is very clear that the structure factor peak and oscillations are the contributions of core-shell LDL particles. On the other hand comparison of USAXS profiles of full yolk and yolk-granules confirm that the Porod law ($I(q) \propto q^{-4}$) behaviour at low-q is the contribution of surface scattering of micron-sized yolk-granules.

6 Supplementary Note 6: Temporal evolution of egg yolk structure during heating

The structural changes that accompany heat-induced non-equilibrium processes in egg yolk are accessed by extracting time-resolved, azimuthally integrated scattering intensity $I(q)$. In the following sections, we show the changes in $I(q)$ of egg yolk when heated to temperatures in the range of (63 °C to 100 °C).

6.1 Structural evolution at low temperatures (63 °C $\leq T <$ 75 °C)

The temporal evolution of $I(q)$ of egg yolk heated to temperatures in the range 63 °C $\leq T <$ 75 °C are consolidated in Supplementary Fig. 10. While the scattering intensity at low-q is unaffected during heating, the $I(q)$ at high-q (above 0.02 nm^{-1}) is increased with absolute waiting time, t'_{w}. In order to quantify these changes in high-q regime, we calculate the scattering invariant (Q) [16, 26] using the definition,

$$Q = \int_{q_1}^{q_2} q^2 \, I(q) \, \mathrm{d}q. \tag{4}$$

The lower and upper limit of integration are $q_1 = 0.02\,\mathrm{nm}^{-1}$ and $q_2 = 0.17\,\mathrm{nm}^{-1}$. The extracted values of Q for $T <$ 75 °C are shown in Supplementary Fig. 11. The error in Q is estimated by considering the standard error in $I(q)$ data points. As shown in Supplementary Fig. 11, Q increases with t'_{w} for all temperatures. The increase in Q during the heating cycle (from room temperature to set temperature) is not appreciable ($t'_{\mathrm{w}} \leq 30$ s). The initial spread in Q between measurements is due to the inherent heterogeneity of the sample volume (as described in Supplementary Fig. 1). Therefore, we perform a normalisation with respect to the initial Q value ($Q_0 = Q$ value at $t'_{\mathrm{w}} = t_{\mathrm{heating}}$ = time taken to reach set temperature from 22 °C), to eliminate the effects of sample heterogeneity on major heat-induced structural changes. In Supplementary Fig. 12 the evolution of Q/Q_0 as a function of isothermal waiting time, t_{w} ($= t'_{\mathrm{w}} - t_{\mathrm{heating}}$) is depicted.

Interestingly, the rate of increase in Q is temperature-dependent. In addition, for any given temperature, the slope of the curve increases with t_{w}. Clearly, there are two regimes (low and high t_{w}) where the slope of the curves

is constant. In order to estimate the transition time of these two regimes, we fit the curves using a power law function ($Q/Q_0 \sim t_{\mathrm{w}}^{\alpha}$). The power law fits at low and high t_{w} are displayed in Supplementary Fig. 12. The intersection point of power-law fits gives t^* for a specific T.

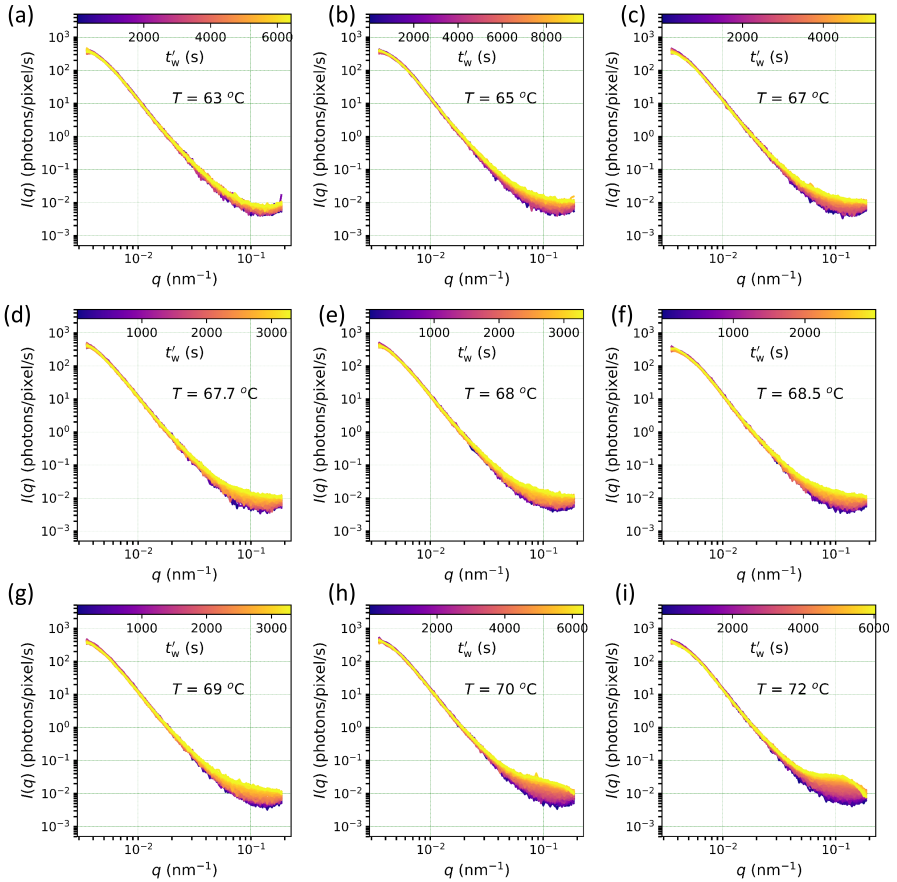

Supplementary Fig. 10 The $I(q)$ collected at the sample temperature of (a) 63 °C, (b) 65 °C, (c) 67 °C, (d) 67.7 °C, (e) 68 °C, (f) 68.5 °C, (g) 69 °C, (h) 70 °C, and (i) 72 °C as a function of t'_{w} as indicated by the scale bar. The emergence of the peak in the high-q regime after a long waiting time in (i) is indicative of the onset of LDL aggregation.

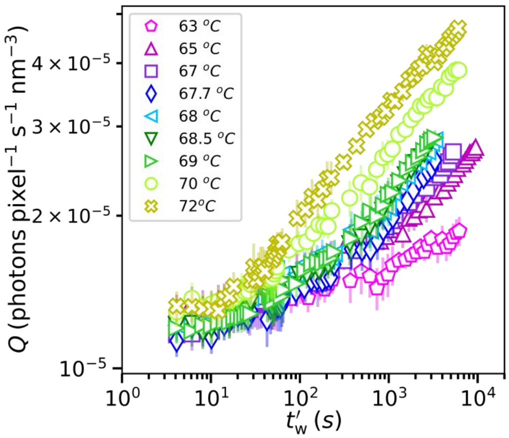

Supplementary Fig. 11 The scattering invariant calculated using Eq. 4 as a function of t'_{w}. The short vertical line close to the x-axis indicates t_{heating}. The colour of the vertical line matches the colour of the data points for a given temperature. The error in Q is estimated by considering the standard error in $I(q)$.

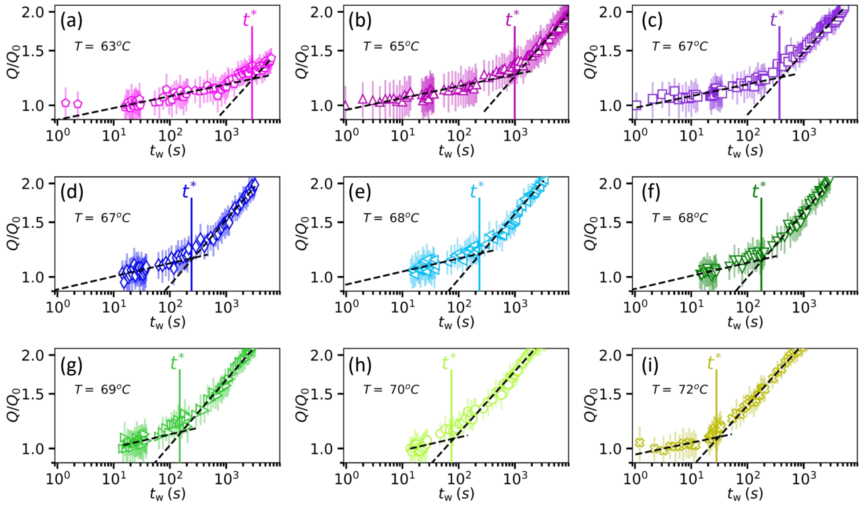

Supplementary Fig. 12 The normalised scattering invariant as a function of t_{w}, for sample temperature of (a) 63 °C, (b) 65 °C, (c) 67 °C, (d) 67.7 °C, (e) 68 °C, (f) 68.5 °C, (g) 69 °C, (h) 70 °C, and (i) 72 °C. The normalisation is performed using $Q_0 = Q(t_{\mathrm{w}} = 0)$. The black dashed lines indicate power law fits (t_{w}^{α}) and the intersection (t^*) of power law fits is indicated by the vertical line. The error in Q is estimated by considering the standard error in $I(q)$.

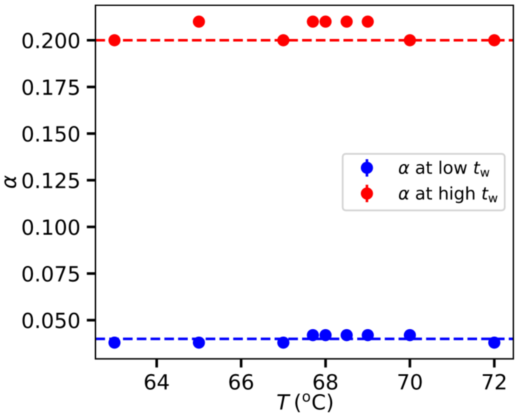

Supplementary Fig. 13 The power law exponent α extracted from the fits shown in Supplementary Fig. 12. The horizontal blue and red dashed lines represent $\alpha = 0.04$ and $\alpha = 0.2$ respectively. The error bars indicate the parameter uncertainty obtained from the fits using least-squares minimization.

6.2 Structure evolution at high temperatures (75 °C ≤ *T* ≤ 100 °C)

The temporal evolution of $I(q)$ of egg yolk heated to temperatures in the range $75\,^{\circ}\mathrm{C} \leq T \leq 100\,^{\circ}\mathrm{C}$ are displayed in Supplementary Fig. 14. The noticeable feature of scattering profiles is the emergence of a broad peak which is shifting to high-length scales with time. At high temperatures, the peak has disappeared at long t'_{w}. There are two possible reasons for this, (a) the aggregates have become considerably larger and are no longer accessible in the experimental q-regime, and (b) the peak is hidden in the high intensity at low-q (Porod scattering of yolk-granules). The peak position (q_{peak}) is extracted from the $I(q)$ (see next section for more details), and characteristic correlation length ζ is determined using the equation,

$$\zeta = \frac{2\pi}{q_{\mathrm{peak}}} \tag{5}$$

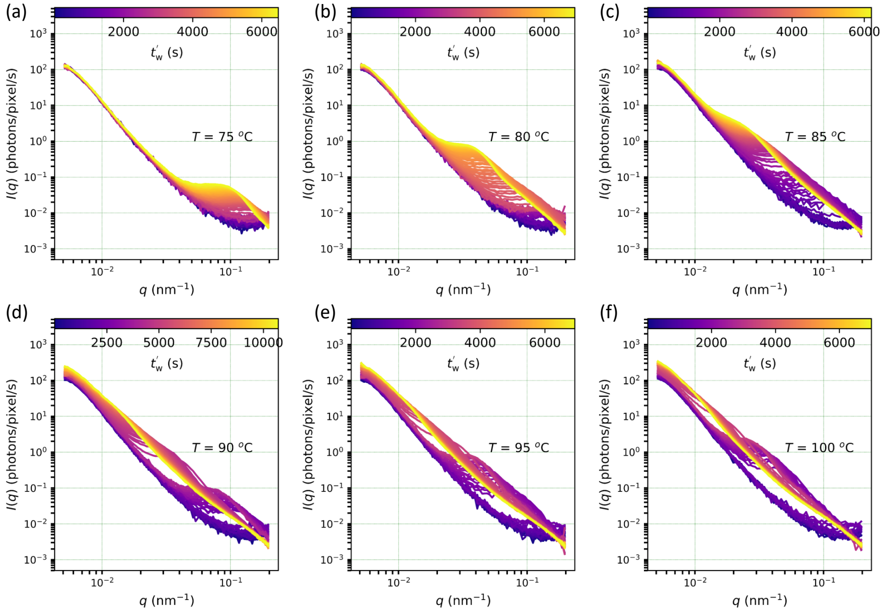

Supplementary Fig. 14 The time-resolved $I(q)$ of egg yolk at a sample temperature of (a) 75 °C, (b) 80 °C, (c) 85 °C, (d) 90 °C, (e) 95 °C, and (f) 100 °C. The colour bars represent t'_{w}.

7 Supplementary Note 7: Extraction of correlation length from scattering intensity

For egg yolk samples, it was difficult to find the peak position from the raw data. Hence we have subtracted the background of $I(q) = Cq^{-\delta}$ to extract the peak position as depicted in Supplementary Fig. 15. After the subtraction of $I(q) = Cq^{-\delta}$ from the raw data, the LDL-aggregate peak was clearly visible. An example illustrating these steps for an egg yolk data is shown in Supplementary Fig. 15. After the subtraction, we used the function peak_prominences from "scipy.signal" Python library to find the peak position as indicated by a red dot in Supplementary Fig. 15. At room temperature, δ was equal to 4, but with heating, there was a slight change in the slope (Supplementary Fig. 14), hence for subtracting the power-law background in the low-q, δ was set free.

Whereas for yolk-plasma samples, where q_{peak} position was clearly visible due to the absence of Porod scattering intensity at low-q regime, we simply extracted the maxima using peak_prominences from "scipy.signal" Python library as illustrated in Supplementary Fig. 16. The error in q_{peak} is estimated from the q resolution at the peak position.

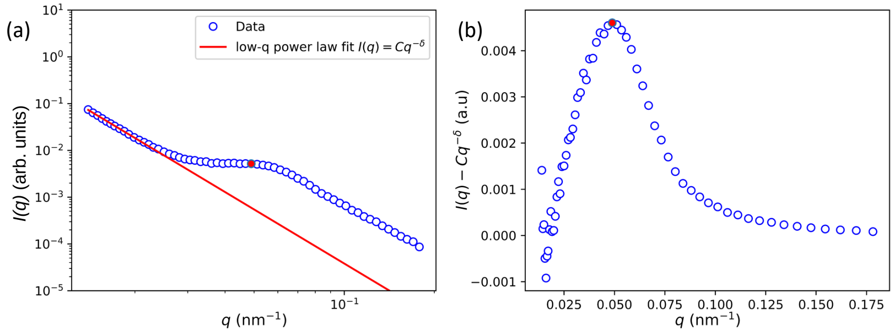

Supplementary Fig. 15 (a) A typical $I(q)$ data of heated egg yolk sample with power-law ($I(q) = Cq^{-\delta}$) fit at low-q regime. (b) The scattering intensity after the subtraction of $Cq^{-\delta}$. The red dot in the plots indicates the q_{peak}.

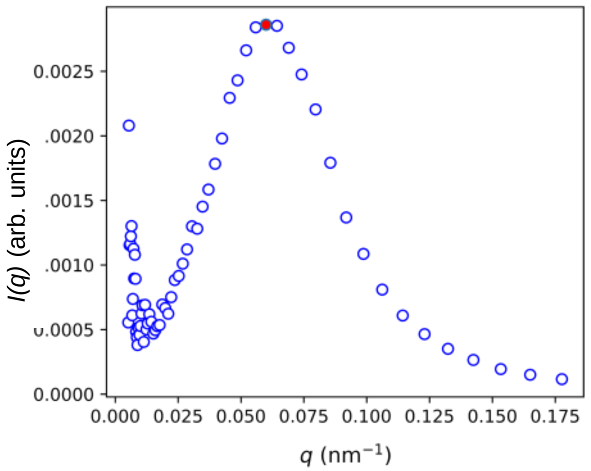

Supplementary Fig. 16 A typical $I(q)$ data of heated egg yolk-plasma sample with the q_{peak} position indicated with a red dot.

The correlation length, ζ estimated for egg yolk and egg yolk-plasma are shown in Supplementary Fig. 17.

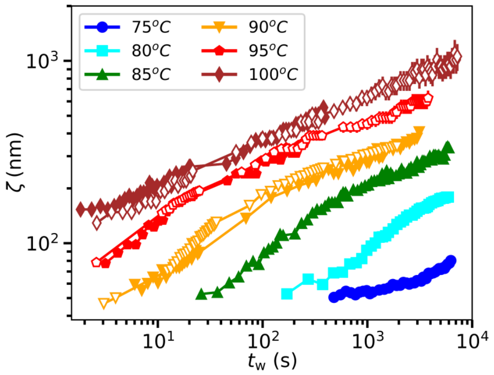

Supplementary Fig. 17 The correlation length ($\zeta = \frac{2\pi}{q_{\text{peak}}}$) estimated from $I(q)$ as a function of t_{w} for egg yolk (solid) and egg yolk-plasma (open). The temperatures are mentioned in the figure legends. The error bars are obtained from the error in q_{peak} estimation (see Supplementary Note 7).

The temporal evolution of SAXS intensity of egg yolk-plasma heated to $T = 85\,°\mathrm{C}$ is depicted in Supplementary Fig. 18. For better clarity, the profiles until $t'_{\mathrm{w}} \approx 32\,\mathrm{s}$ is shown in Supplementary Fig. 18b. Clearly, the position of the structure factor peak is not changed for the first 32 s and interesting changes are observed in the low-q regime, where scattering intensity is increased drastically for a very short period of time, indicating the denaturation of proteins observed in Supplementary Fig. 10.

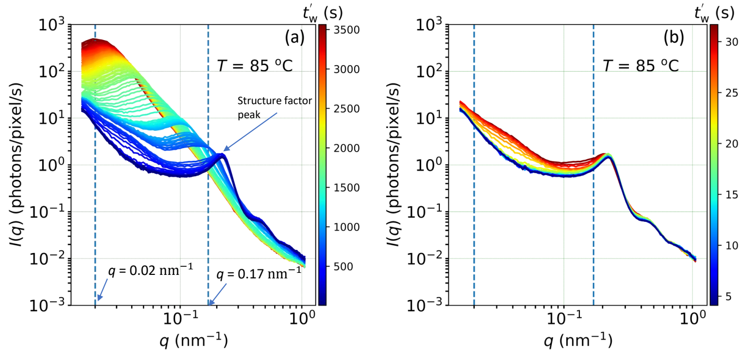

Supplementary Fig. 18 The temporal evolution of SAXS profiles of egg yolk-plasma collected at the sample temperature of $85\,°\mathrm{C}$ for t'_{w} (a) 0 - 3500 s (b) 0 - 32 s. The t'_{w} is indicated by the scale bar. The vertical lines corresponds to $q = 0.02\,\mathrm{nm}^{-1}$ and $q = 0.17\,\mathrm{nm}^{-1}$. The structure factor peak at $q = 0.22\,\mathrm{nm}^{-1}$ give the average distance ($= \frac{2\pi}{q} \approx 30$ nm) between close by LDLs. This data is collected at P10 beamline, PETRA III, Hamburg.

7.1 Internal structure of LDL aggregates

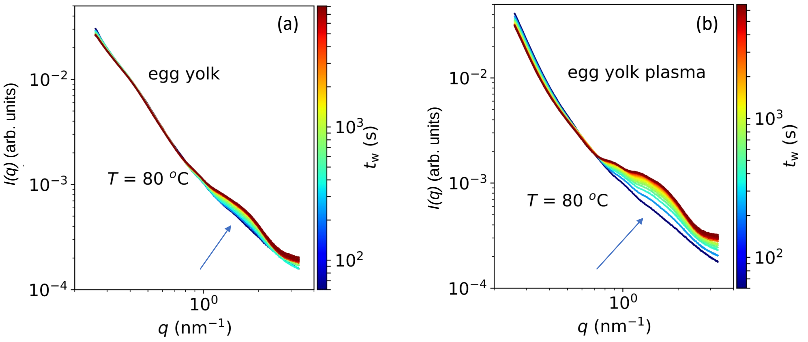

Supplementary Fig. 19 The temporal evolution of SAXS intensity of (a) egg yolk, and (b) egg yolk-plasma heated to $80\,°\mathrm{C}$ as a function of waiting time as indicated by the scale bar. The arrow points to the emergence of a broad peak at $q \approx 1.8\,\mathrm{nm}^{-1}$. This data is collected at beamline BL2 of the DELTA synchrotron radiation source.

To investigate the internal structure of LDL aggregates, we perform scattering measurements at the small-angle and wide-angle X-ray scattering (SAXS and WAXS) beamline BL2 of the DELTA synchrotron radiation source. Details of measurement are provided in Supplementary Fig. 4. The emergence of a broad peak at $q \approx 1.8\,\mathrm{nm}^{-1}$ (Supplementary Fig. 19), is indicative of the formation of intra-aggregate structures. We speculate that this implies the formation of micellar structures inside the LDL aggregates. But, future investigations are required to confirm this hypothesis

8 Supplementary Note 8: Dynamics of egg yolk and its constituents

8.1 Equilibrium dynamics of egg yolk at room temperature

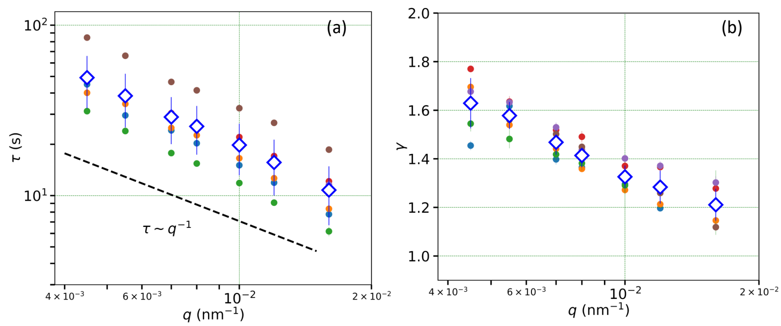

Supplementary Fig. 20 (a) Room temperature relaxation time and (b) KWW exponent (from the fit using Eq. 2) of egg yolk extracted from the same egg and different eggs. Different closed symbol colours represent individual XPCS scans on different egg yolk samples. The dashed lines indicate the power law model $\tau \sim q^{-1}$. The open symbol in (a) and (b) represent the average τ and KWW exponent respectively. The error bars in individual τ and γ curves indicate the parameter uncertainty obtained from the fits using least-squares minimization. The error bars in average τ and γ indicate the standard deviation.

8.2 Egg yolk: TTCs and $|f(q,t)|^2$ during heating

Following the analysis procedure described in Supplementary Fig. 4.2, TTCs and g_2 functions are extracted as a function of absolute waiting time. The temporal evolution of TTC for low temperatures ($63\,^\circ\mathrm{C} \leq T < 75\,^\circ\mathrm{C}$) and high temperatures ($75\,^\circ\mathrm{C} \leq T \leq 100\,^\circ\mathrm{C}$) are depicted in Supplementary Fig. 21 and Supplementary Fig. 23 respectively. For clarity, TTCs are zoomed until $t'_\mathrm{w} = 160$ s and shown in Supplementary Fig. 22 and Supplementary Fig. 24. The $|f(q,t)|^2$ (along with fits using Eq. 2) for low and high T are depicted in Supplementary Fig. 25 and Supplementary Fig. 26 respectively.

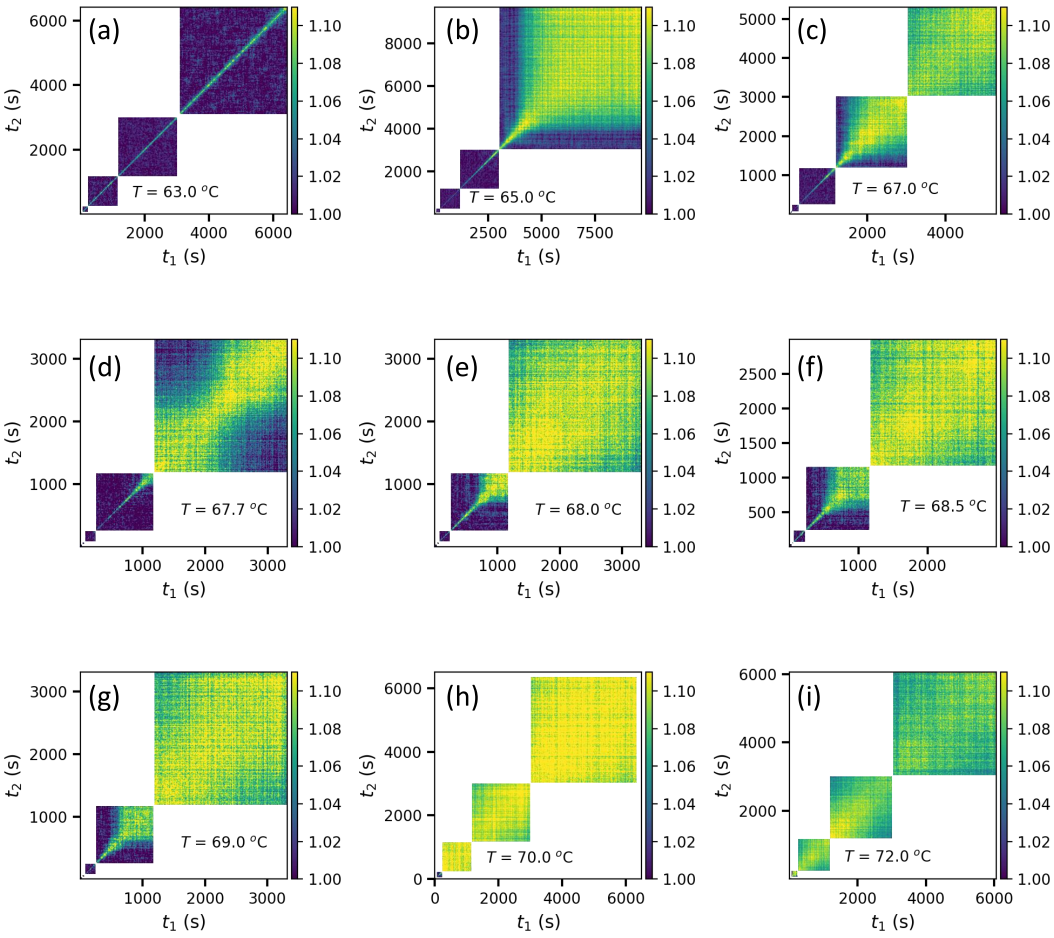

Supplementary Fig. 21 The temporal evolution of TTC for egg yolk heated to (a) 63 °C, (b) 65 °C, (c) 67 °C, (d) 67.7 °C, (e) 68 °C, (f) 68.5 °C, (g) 69 °C, (h) 70 °C, and (i) 72 °C. The TTCs are extracted at $q \approx 0.0055\,\mathrm{nm}^{-1}$.

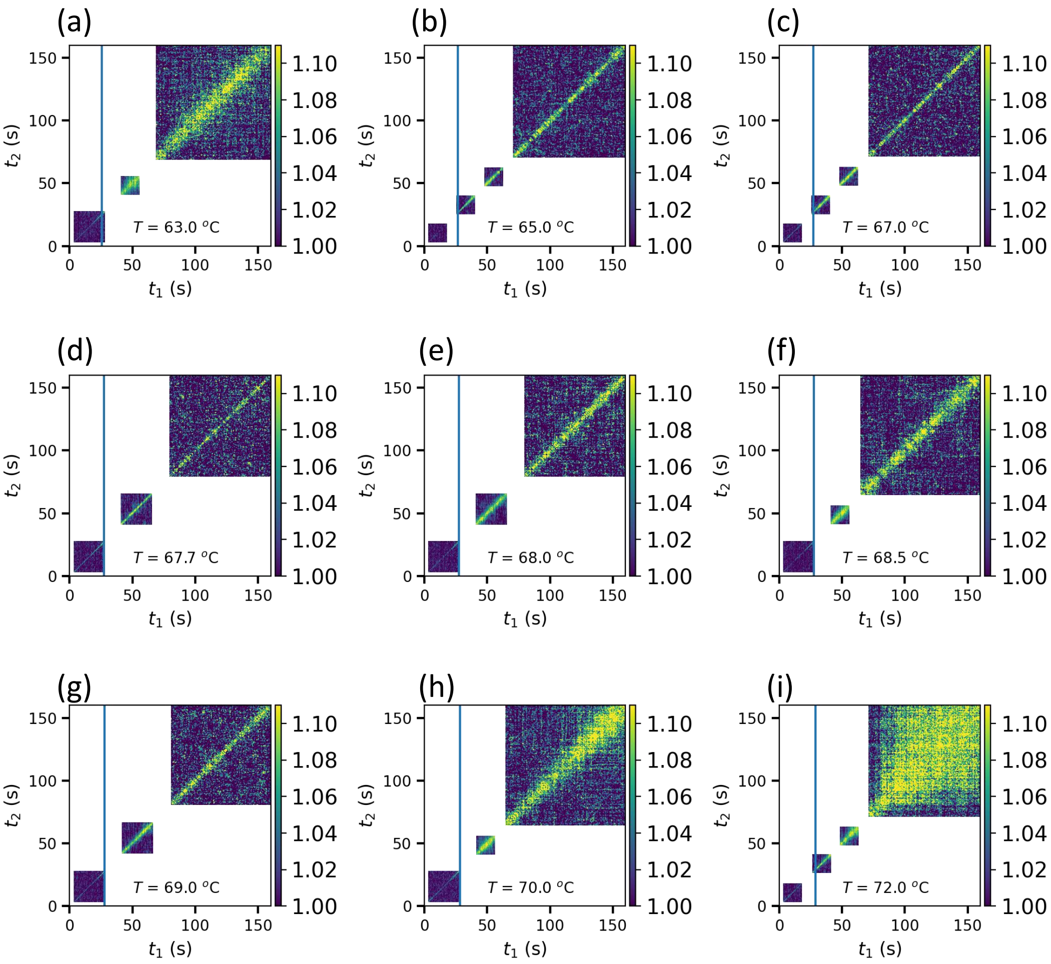

Supplementary Fig. 22 The temporal evolution of TTC for egg yolk heated to temperatures in the range $63\,^{\circ}\mathrm{C} \leq T < 75\,^{\circ}\mathrm{C}$, until $t'_{\mathrm{w}} = 160$ s. The temperatures are mentioned in the respective TTCs. The TTCs are extracted at $q \approx 0.0055\,\mathrm{nm}^{-1}$. The vertical line in each plot corresponds to respective t_{heating}.

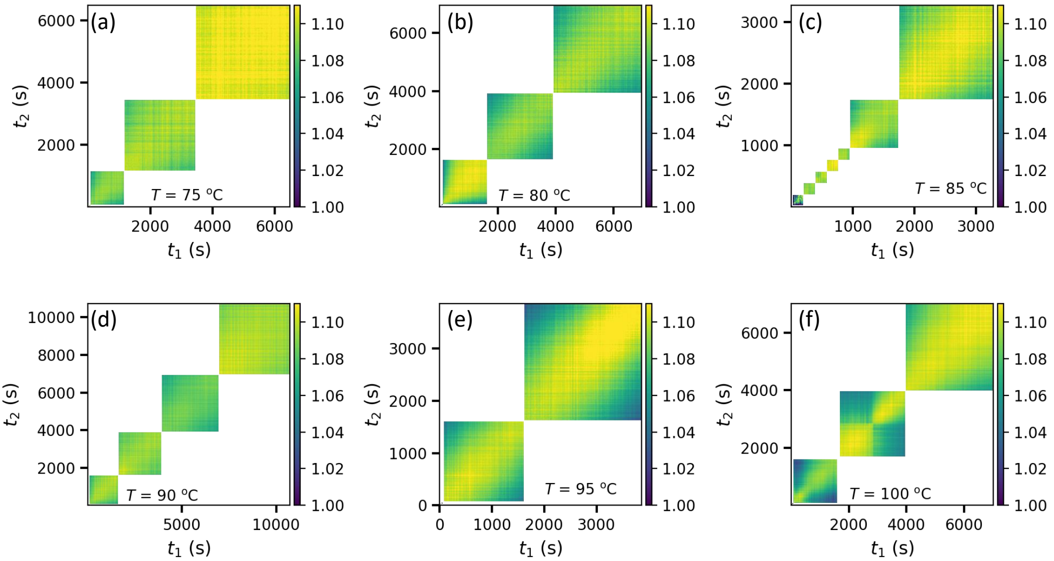

Supplementary Fig. 23 The temporal evolution of TTC for egg yolk heated to (a) 75 °C, (b) 80 °C, (c) 85 °C, (d) 90 °C, (e) 95 °C, and (f) 100 °C. The TTCs are extracted at $q \approx 0.0055\,\mathrm{nm}^{-1}$.

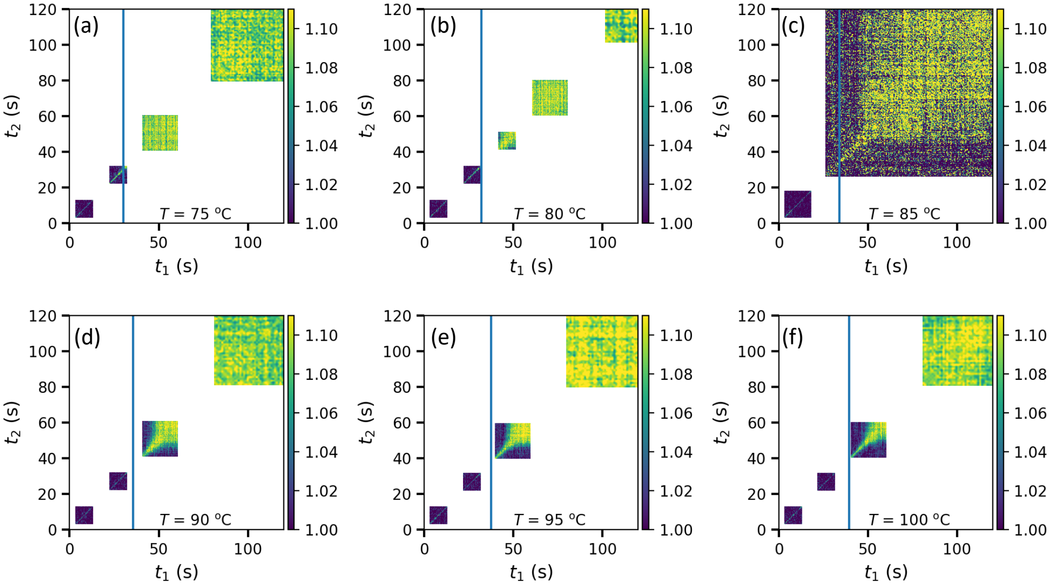

Supplementary Fig. 24 The temporal evolution of TTC for egg yolk heated to temperatures in the range $75\,^{\circ}\mathrm{C} \leq T \leq 100\,^{\circ}\mathrm{C}$, until $t'_{\mathrm{w}} = 160$ s. The temperatures are mentioned in the respective TTCs. The TTCs are extracted at $q \approx 0.0055\,\mathrm{nm}^{-1}$. The vertical line in each plot corresponds to respective t_{heating}.

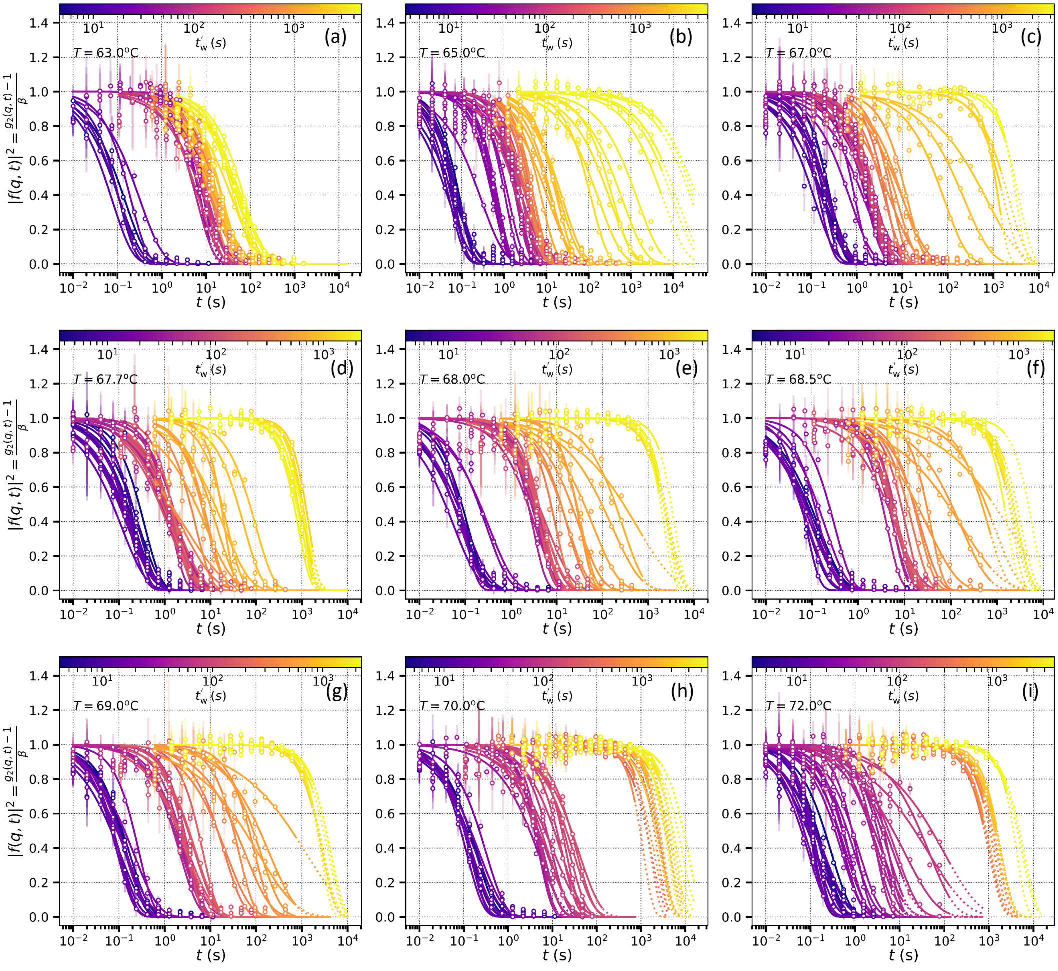

Supplementary Fig. 25 $|f(q,t)|^2$ extracted from TTCs shown in Supplementary Fig. 21) at different waiting times as indicated by the colour bar. The solid curves are fit using Eq. 2. The error bars represent the standard error over TTC lines within a horizontal cut.

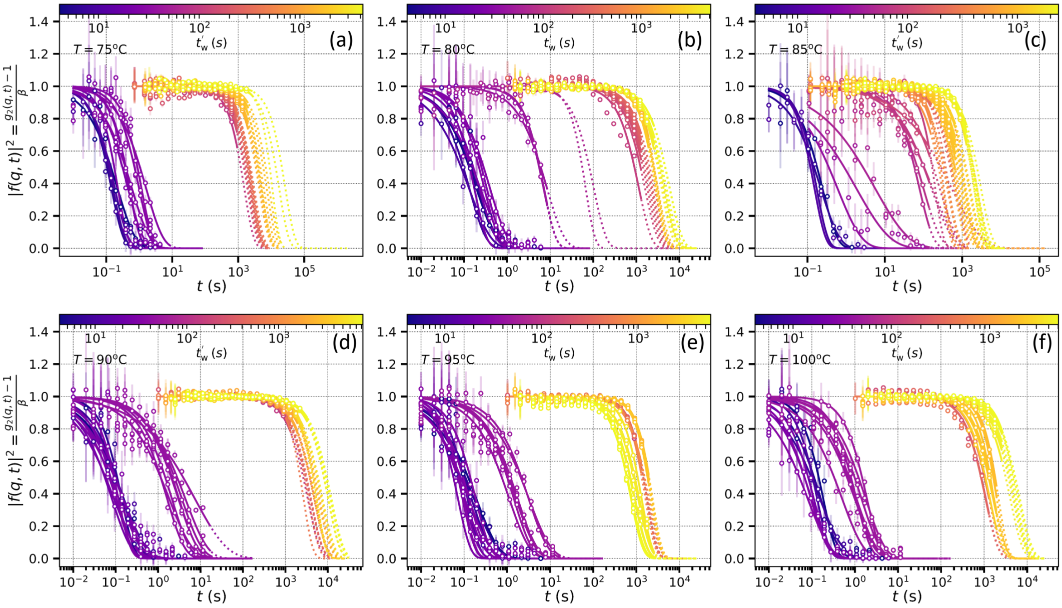

Supplementary Fig. 26 $|f(q,t)|^2$ extracted from TTCs shown in Supplementary Fig. 23 at different waiting times as indicated by the colour bar. The solid curves are fit using Eq. 2. The error bars represent the standard error over TTC lines within a horizontal cut.

8.3 Egg yolk-plasma and egg yolk-granules: TTCs and $|f(q,t)|^2$ during heating

To understand the functional contribution of various constituents to heat-induced non-equilibrium processes in egg yolk, we perform XPCS measurements on egg yolk-plasma and egg yolk-granules which are separated from egg yolk as described in Supplementary Fig. 2. The temporal evolution of TTC for yolk-plasma and yolk-granules are depicted in Supplementary Fig. 27 and Supplementary Fig. 28 respectively. From the TTCs, the $|f(q,t)|^2$ is extracted by following the analysis procedure explained in Supplementary Fig. 4.2 and is shown in Supplementary Fig. 29.

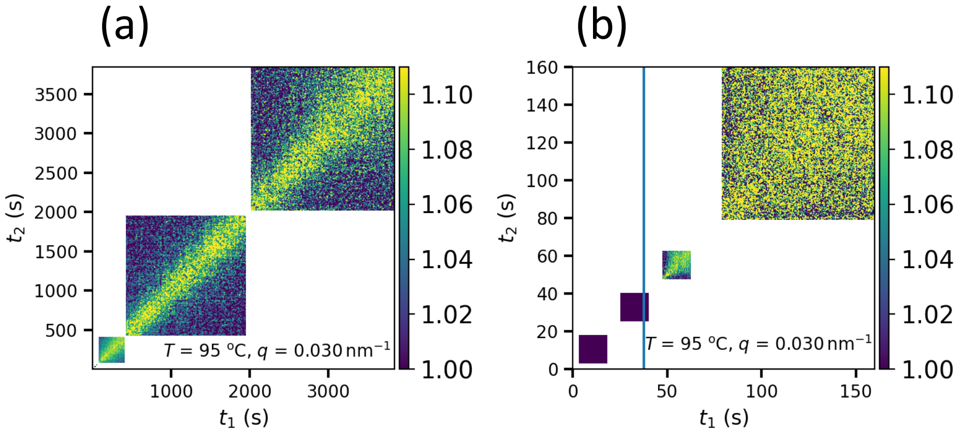

Supplementary Fig. 27 The temporal evolution of TTC for egg yolk-plasma heated to 95 °C for (a) full waiting time of the experiment, (b) until t'_w = 100 s. The TTCs are extracted at $q \approx 0.03\,\mathrm{nm}^{-1}$. The vertical line in (b) corresponds to t_{heating}.

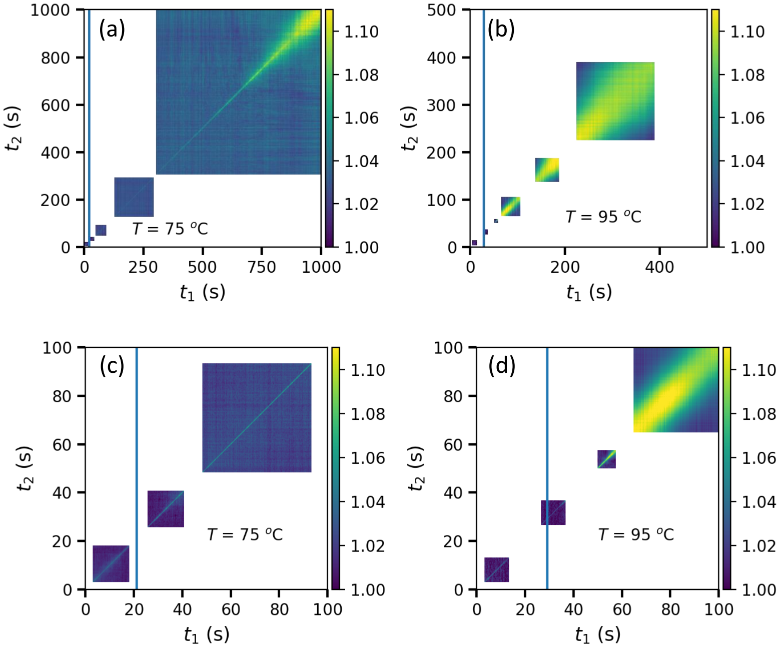

Supplementary Fig. 28 The temporal evolution of TTC for egg yolk-granules heated to (a) 75 °C (b) 95 °C for full waiting time of the experiment. For clarity, (a) and(b) are zoomed until $t'_{\mathrm{w}} = 100$ s and given in (c) and (d) respectively. The TTCs are extracted at $q \approx 0.0055\,\mathrm{nm}^{-1}$. The vertical lines in each plot correspond to respective t_{heating}.

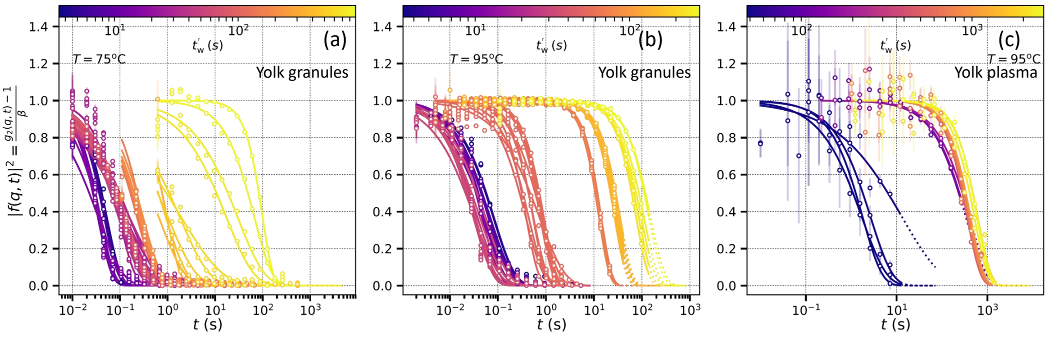

Supplementary Fig. 29 $|f(q,t)|^2$ extracted from TTCs (Supplementary Fig. 28) of egg yolk-granules heated to (a) 75 °C and (b) 95 °C respectively. (c) $|f(q,t)|^2$ extracted from TTCs (Supplementary Fig. 27) of egg yolk-plasma heated to 95 °C. Waiting time is indicated by the colour bar. The solid curves are fit using Eq. 2. The error bars represent the standard error over TTC lines within a horizontal cut.

9 Supplementary Note 9: Egg yolk-plasma: estimation of τ at low-q via extrapolation

As depicted in Supplementary Fig. 30, for egg yolk-plasma samples, the dynamical information for a given q was only accessible when the aggregate peak has moved to that particular q region. For example, dynamical information is only accessible in the fifth and third TTC for $q \approx 0.0055\,\mathrm{nm}^{-1}$ and $q \approx 0.03\,\mathrm{nm}^{-1}$ respectively, when the aggregate peak has shifted to these q values. This means dynamical information at $q \approx 0.0055\,\mathrm{nm}^{-1}$ is only accessible after a long waiting time of $\approx$ 1000 s. To solve this issue we extrapolate the dynamical information at $q \approx 0.03\,\mathrm{nm}^{-1}$ to $q \approx 0.0055\,\mathrm{nm}^{-1}$ by following the relationship $\tau \propto \frac{1}{q}$ as depicted in Supplementary Fig. 31.

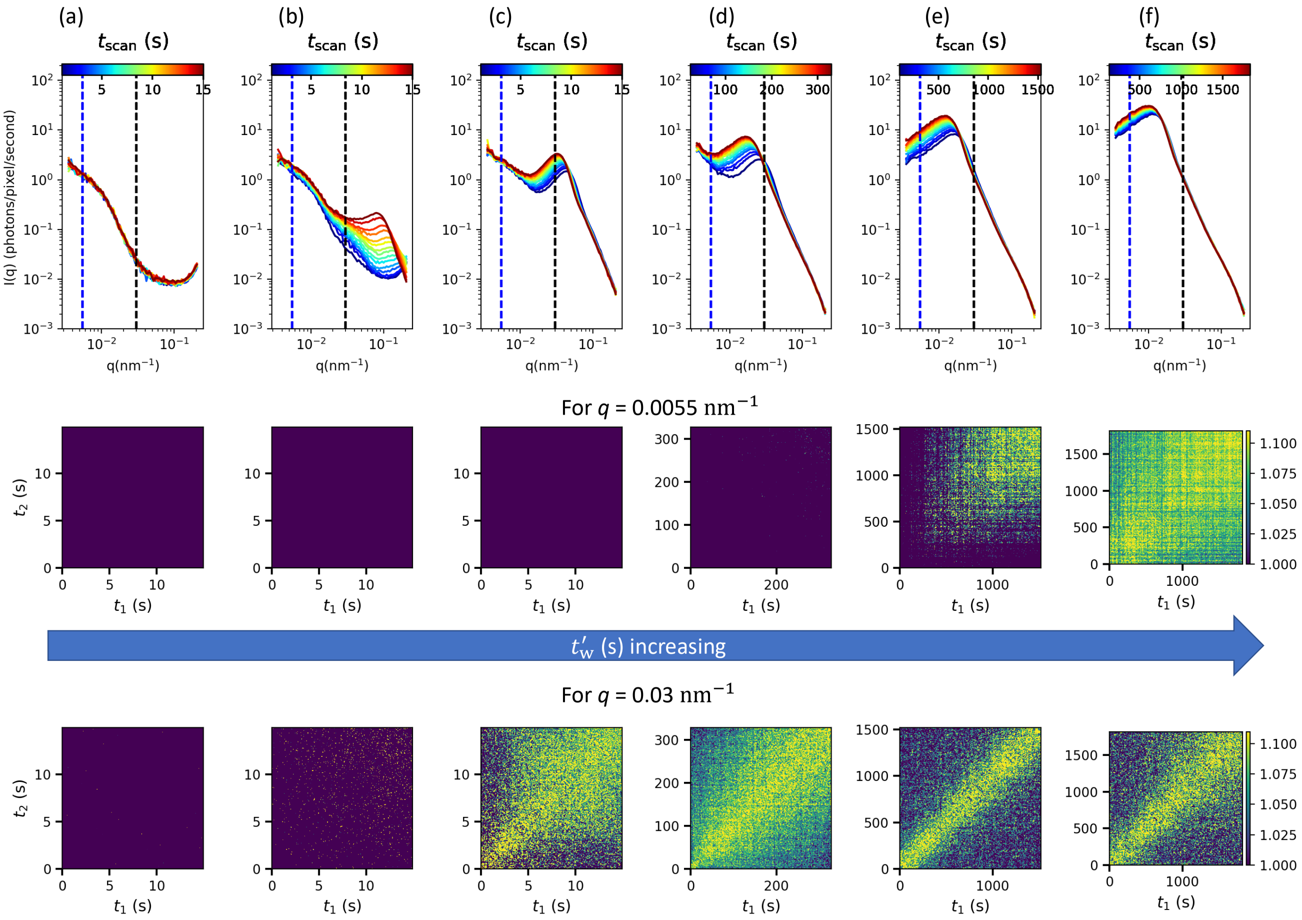

Supplementary Fig. 30 Top row: (a-f) $I(q)$ of egg yolk-plasma heated to 95 °C. (a) to (f) represent successive scans collected in the increasing order of waiting time. The blue and black dashed lines represent $q \approx 0.0055\,\mathrm{nm}^{-1}$ and $q \approx 0.03\,\mathrm{nm}^{-1}$ respectively. The waiting time is indicated by the colour bar. TTCs extracted at $q \approx 0.0055\,\mathrm{nm}^{-1}$ (middle row) and at $q \approx 0.03\,\mathrm{nm}^{-1}$ (bottom row).

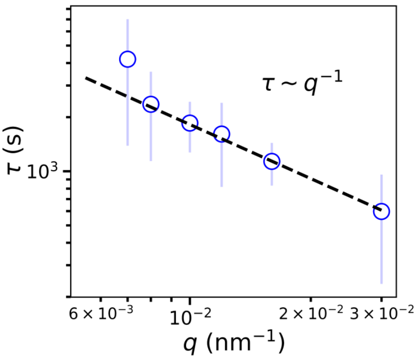

Supplementary Fig. 31 The wave vector dependent dynamics of egg yolk-plasma collected at $T = 95\,^\circ\mathrm{C}$ at a waiting time of $t'_\mathrm{w} \approx 960$ s. The error bars in τ indicate the parameter uncertainty obtained from the fits using least-squares minimization.

9.1 The temporal evolution of relaxation time during heating

The temporal evolution of τ for egg yolk heated to $63\,^{\circ}\mathrm{C} \leq T \leq 100\,^{\circ}\mathrm{C}$ is shown in Supplementary Fig. 32a. The effect of heating time on dynamics is eliminated by normalising the waiting time with respect to t_{heating} (Supplementary Fig. 32b).

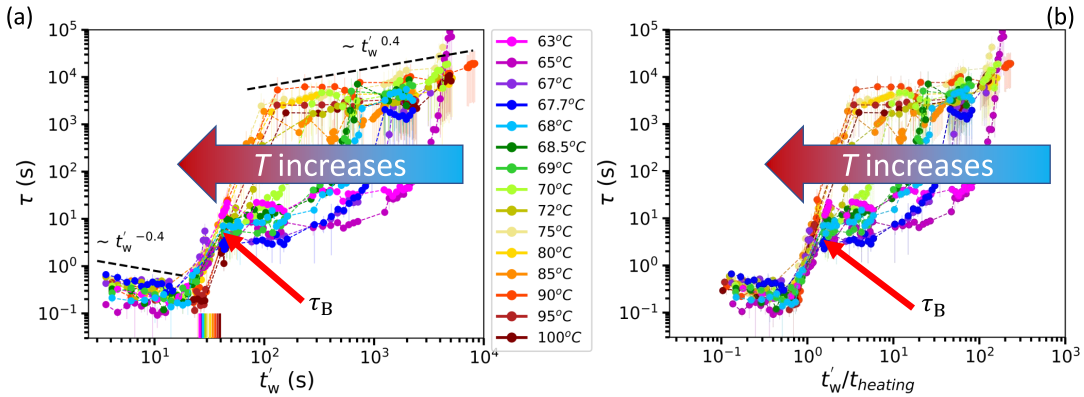

Supplementary Fig. 32 The extracted relaxation times as a function of (a) t'_{w} and (b) the normalised waiting time, $t'_{\mathrm{w}}/t_{\mathrm{heating}}$, where t_{heating} is the time taken for reaching the set temperature from $22\,^{\circ}\mathrm{C}$. The temperatures are mentioned in the legends. The dashed lines represent power law fits and the power law exponents are mentioned in the figure. The error bars in τ indicate the parameter uncertainty obtained from the fits using least-squares minimization.

9.2 Comparison of apparent viscosity from viscometry and relaxation time from XPCS

The in-situ viscometry measurements were performed using a Bolin Gemini rotational HR nano-rheometer. The egg yolk sample was heated to a set temperature T from $20\,^{\circ}\mathrm{C}$ and the apparent viscosity η at a shear rate of $10\,\mathrm{s}^{-1}$ was measured in parallel. This shear rate is within the reported ranges for swallowing shear rates [27] and hence can be directly correlated with the mouth feel of food. The in-situ measurements were performed in a temperature range of 64–$69\,^{\circ}\mathrm{C}$. In Supplementary Fig. 33 we compare the apparent viscosity at different temperatures. Interestingly, the temporal evolution of η displays four regimes similar to our XPCS data. Initially, the viscosity of egg yolk is almost constant with $\eta \approx 2\,\mathrm{Pa\,s}$ for ≈ 30 s (regime-A). During this time period, the temperature of the yolk increases from $20\,^{\circ}\mathrm{C}$ to set T. After reaching this set temperature, a linear increase in viscosity can be observed. This continues until η reaches a value between $20\,\mathrm{Pa\,s}$ (for $64\,^{\circ}\mathrm{C}$) to $40\,\mathrm{Pa\,s}$ (for $69\,^{\circ}\mathrm{C}$). After this, the change in the viscosity is still linear with time

(regime-B) but with a slow rate until a characteristic time, t^*_{visco}, beyond which the viscosity starts to increase exponentially (regime-C). Clearly, this sharp increase in η indicates the sol-gel transition [28] and t^*_{visco} is the sol-gel transition time. Interestingly, we obtain also a master curve upon normalising t_{w} with t^*_{visco} (Supplementary Fig. 33b), which is very similar to the master curve obtained for τ (Supplementary Fig. 33c). Moreover, the sol-gel transition times, t^* from XPCS and viscometry (t^*_{visco}) are in very good agreement (Supplementary Fig. 33d).

However, there are a few differences in the τ and η data in the initial waiting time after regime-A. In the XPCS data, we see a sudden transition of the system from regime-A to regime-B while there is a progressive change in the viscosity data. This may arise from the change in the thermal history of the sample. For the XPCS measurements, the samples are contained in a thin capillary of diameter $\approx$ 1.5 mm, whereas for the viscometry measurements, the samples are spread on a plate and the time needed to reach the same temperature everywhere in the sample may be slightly higher than the XPCS case. This might be the reason behind the observed differences between regime-A and regime-B.

We can not resolve the viscosity changes during the sol-gel transition time at temperatures above 69 °C as this transition is too fast and already occurring while the whole sample reaches the final temperature set point.

9.3 Comparison of microstructure from SEM with XPCS results

The properties of food texture are usually characterised by means of the mechanical and/or geometrical characteristic properties [29]. The grainy microstructure of food samples can be characterized by the size and shape of the sample's constituents [29] for example by using microscopy techniques [30]. Here, we employ ex-situ scanning electron microscopy to investigate the effect of temperature on the grainy microstructure of heated egg yolk.

In Supplementary Fig. 34(a-c) we show SEM images collected from egg yolk heated to temperatures in the range 90-100 °C for 300 s. The grainy microstructure of egg yolk is indeed apparent in the SEM images. The size of these aggregates increases with increasing temperature (Supplementary Fig. 34d). Importantly, we observe that the overall evolution of sizes (from SEM) and correlation lengths (from XPCS) of the food microstructure as a function of temperature are in good agreement as depicted in Supplementary Fig. 34d.

However, it was difficult to perform SEM imaging of yolk samples heated at lower temperatures for the same waiting time due to aggregate sizes being smaller than 200 nm. To resolve such small structures, we had to expose the sample for a long time (compared to imaging bigger aggregates at high temperatures) to the electron beam (focusing, optimization of contrast etc.

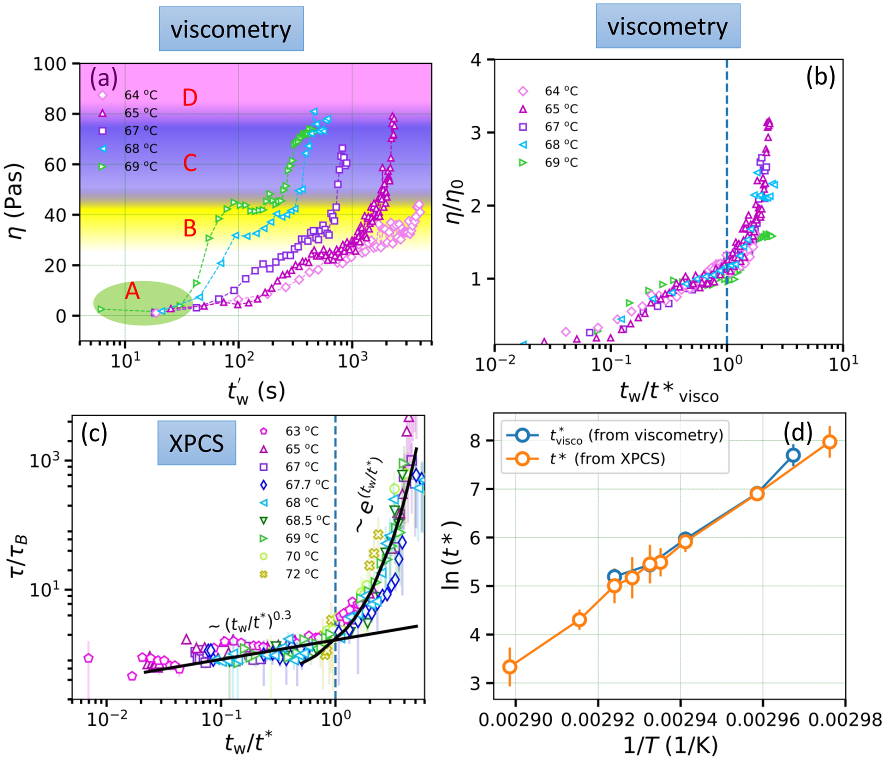

Supplementary Fig. 33 (a) Apparent viscosity η of egg yolk heated to temperatures in the range of 64-69 °C as a function of absolute waiting time. Regime-A, B, C and D are indicated with the background colours of green, yellow, blue and pink respectively (similar to Fig. 5a of the manuscript). (b) Normalised apparent viscosity with respect to $\eta_0 \approx \eta(t = t^*_{\text{visco}})$, as a function of isothermal waiting time, t_{w} normalised with respect to t^*_{visco}. (c) Master plot of τ from XPCS. The error bars in τ indicate the parameter uncertainty obtained from the fits using least-squares minimization. (d) Comparison of sol-gel transition time from XPCS and viscometry. The error bar in t^* is estimated using the error in the fit parameters of power law fits of Q/Q_0 at low-q and high-q regime (see Supplementary Note 6). The error in t^*_{visco} derived from the time resolution in (a).

is necessary to resolve smaller structures). This led to imaging artefacts at low temperatures preventing us from quantitative analysis. We did not use any chemical fixation techniques to improve the electronic contrast, as it can damage the lipid content of egg yolk [31].

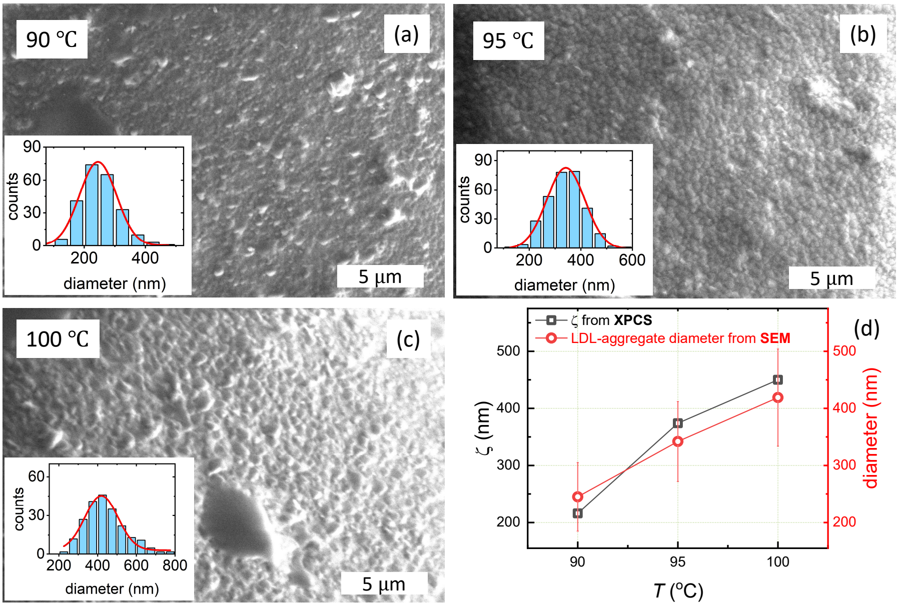

Supplementary Fig. 34 SEM images of egg yolk collected at room temperature after being heated at (a) 90 °C, (b) 95 °C, and (c) 100 °C for 300 s. Inset shows the histogram of LDL aggregate diameter. (d) LDL aggregate correlation length ζ from XPCS and aggregate diameter from SEM as a function of temperature. Error in LDL-aggregate diameter is the standard error from the Gaussian fits shown in (a-c) insets. The error bars of ζ are obtained from the error in q_{peak} estimation (see Supplementary Note 7).

Supplementary References

[1] Mann, K. & Mann, M. The chicken egg yolk plasma and granule proteomes. *Proteomics* **8** (1), 178–191 (2008) .

[2] Anton, M. Egg yolk: structures, functionalities and processes. *Journal of the Science of Food and Agriculture* **93** (12), 2871–2880 (2013) .

[3] https://www.biorender.com/.

[4] Nakamura, R., Hayakawa, M. & Sato, Y. Isolation and fractionation of the protein moiety of egg yolk low density lipoprotein. *Poultry Science* **56** (4), 1148–1152 (1977) .

[5] Reiser, M. & Rosca, R. https://github.com/reiserm/Xana.

[6] Perakis, F. & Gutt, C. Towards molecular movies with X-ray photon correlation spectroscopy. *Physical Chemistry Chemical Physics* **22** (35), 19443–19453 (2020) .

[7] Begam, N. *et al.* Kinetics of network formation and heterogeneous dynamics of an egg white gel revealed by coherent X-ray scattering. *Physical Review Letters* **126** (9), 098001 (2021) .

[8] Möller, J., Sprung, M., Madsen, A. & Gutt, C. X-ray photon correlation spectroscopy of protein dynamics at nearly diffraction-limited storage rings. *International Union of Crystallography Journal* **6** (5), 794–803 (2019) .

[9] Lehmkühler, F., Roseker, W. & Grübel, G. From femtoseconds to hours–measuring dynamics over 18 orders of magnitude with coherent X-rays. *Applied Sciences* **11** (13), 6179 (2021) .

[10] Zhang, Q., Dufresne, E. M. & Sandy, A. R. Dynamics in hard condensed matter probed by X-ray photon correlation spectroscopy: Present and beyond. *Current Opinion in Solid State and Materials Science* **22** (5), 202–212 (2018) .

[11] Bikondoa, O. On the use of two-time correlation functions for X-ray photon correlation spectroscopy data analysis. *Journal of Applied Crystallography* **50** (2), 357–368 (2017) .

[12] Madsen, A., Leheny, R. L., Guo, H., Sprung, M. & Czakkel, O. Beyond simple exponential correlation functions and equilibrium dynamics in X-ray photon correlation spectroscopy. *New Journal of Physics* **12** (5), 055001 (2010) .

[13] Shpyrko, O. G. X-ray photon correlation spectroscopy. *Journal of Synchrotron Radiation* **21** (5), 1057–1064 (2014) .

[14] Girelli, A. *et al.* Microscopic dynamics of liquid-liquid phase separation and domain coarsening in a protein solution revealed by X-ray photon correlation spectroscopy. *Physical Review Letters* **126** (13), 138004 (2021) .

[15] Timmermann, S. *et al.* Automated matching of two-time X-ray photon correlation maps from phase-separating proteins with cahn–hilliard-type simulations using auto-encoder networks. *Journal of Applied Crystallography* **55** (4) (2022) .

[16] Moron, M. *et al.* Gelation dynamics upon pressure-induced liquid–liquid phase separation in a water–lysozyme solution. *The Journal of Physical Chemistry B* **126** (22), 4160–4167 (2022) .

[17] Ragulskaya, A. *et al.* Reverse-engineering method for XPCS studies of non-equilibrium dynamics. *International Union of Crystallography Journal* **9** (4) (2022) .

[18] Lehmkühler, F. *et al.* Emergence of anomalous dynamics in soft matter probed at the european XFEL. *Proceedings of the National Academy of Sciences* **117** (39), 24110–24116 (2020) .

[19] Reiser, M. *et al.* Resolving molecular diffusion and aggregation of antibody proteins with megahertz X-ray free-electron laser pulses. *Nature Communications* **13**, 2041–1723 (2022) .

[20] Williams, G. & Watts, D. C. Non-symmetrical dielectric relaxation behaviour arising from a simple empirical decay function. *Transactions of the Faraday Society* **66**, 80–85 (1970) .

[21] Ruta, B. *et al.* Hard X-rays as pump and probe of atomic motion in oxide glasses. *Scientific Reports* **7** (1), 1–8 (2017) .

[22] Pintori, G., Baldi, G., Ruta, B. & Monaco, G. Relaxation dynamics induced in glasses by absorption of hard X-ray photons. *Physical Review B* **99** (22), 224206 (2019) .

[23] Chushkin, Y. *et al.* Probing cage relaxation in concentrated protein solutions by X-ray photon correlation spectroscopy. *Physical Review Letters* **129**, 238001 (2022) .

[24] Meuer, H.-J. & Egbers, C. Changes in density and viscosity of chicken egg albumen and yolk during incubation. *Journal of Experimental Zoology* **255** (1), 16–21 (1990) .

[25] Anitas, E. M. in *Small-angle scattering from mass and surface fractals* (ed.López-Ruiz, R.) *Complexity in Biological and Physical Systems* Ch. 10 (IntechOpen, Rijeka, 2017).

[26] Da Vela, S. *et al.* Interplay between glass formation and liquid–liquid phase separation revealed by the scattering invariant. *The Journal of Physical Chemistry Letters* **11** (17), 7273–7278 (2020) .

[27] Pollen, N., Daubert, C. R., Prabhasankar, P., Drake, M. & Gumpertz, M. Quantifying fluid food texture. *Journal of texture studies* **35** (6), 643–657 (2004) .

[28] Innocenzi, P. *et al. The sol to gel transition* (Springer, 2016).

[29] Szczesniak, A. S. Classification of textural characteristics a. *Journal of food science* **28** (4), 385–389 (1963) .

[30] Dong, J. & St. Jeor, V. L. in *Food microstructure techniques* (ed.Nielsen, S. S.) *Food Analysis* 557–570 (Springer International Publishing, Cham, 2017).

[31] Hsu, K.-C., Chung, W.-H. & Lai, K.-M. Histological structures of native and cooked yolks from duck egg observed by sem and cryo-sem. *Journal of agricultural and food chemistry* **57** (10), 4218–4223 (2009) .
